# Peritoneal Tuberculosis in a Pregnant Woman from Haiti, United States

**DOI:** 10.3201/eid1903.121109

**Published:** 2013-03

**Authors:** Kevin L. Ard, Brian T. Chan, Danny A. Milner, Paul E. Farmer, Serena P. Koenig

**Affiliations:** Affiliations: Brigham and Women’s Hospital, Boston, Massachusetts, USA (K.L. Ard, B.T. Chan, D.A. Milner Jr, P.E. Farmer, S.P. Koenig);; Massachusetts General Hospital, Boston (K.L. Ard, B.T. Chan)

**Keywords:** peritonitis, tuberculous, pregnancy, tuberculosis, tuberculosis and other mycobacteria, Haiti

**To the Editor:** A 29-year-old woman at 23 weeks’ gestation during her first pregnancy came to our hospital’s obstetrics clinic after 6 days of vaginal bleeding and abdominal pain. She had not experienced fever, sweats, weight loss, contractions, or other symptoms. She was otherwise healthy; she was taking no medications, but was taking iron and multivitamin supplements. She had legally immigrated to the United States from Haiti 8 months previously and had no known tuberculosis contacts. Physical examination disclosed brown vaginal discharge and a closed cervix. Obstetric ultrasound was normal, and vaginal swab samples were negative for *Neisseria gonorrhea* and *Chlamydia trachomatis*.

Over the ensuing 2 weeks, her vaginal bleeding and abdominal pain worsened. She was admitted to the hospital. Physical examination revealed vaginal bleeding, but her condition was otherwise unchanged. Routine laboratory studies were normal. Repeat obstetric ultrasound showed a viable fetus, ascites, and a 15 × 15 × 3–cm rind of echogenic material anterior to the uterus. This abnormality was in the upper abdomen, an area not imaged on her previous ultrasound. Abdominal magnetic resonance imaging revealed moderate ascites and a 21 × 14 × 3–cm omental mass of intermediate intensity on T1 and T2 sequences; there was no lymphadenopathy (Figure). A tiny left pleural effusion was seen on chest radiograph. Routine HIV and tuberculin skin test results had been negative 4 months previously, and pre-immigration examination results and chest radiograph had been normal.

**Figure:**
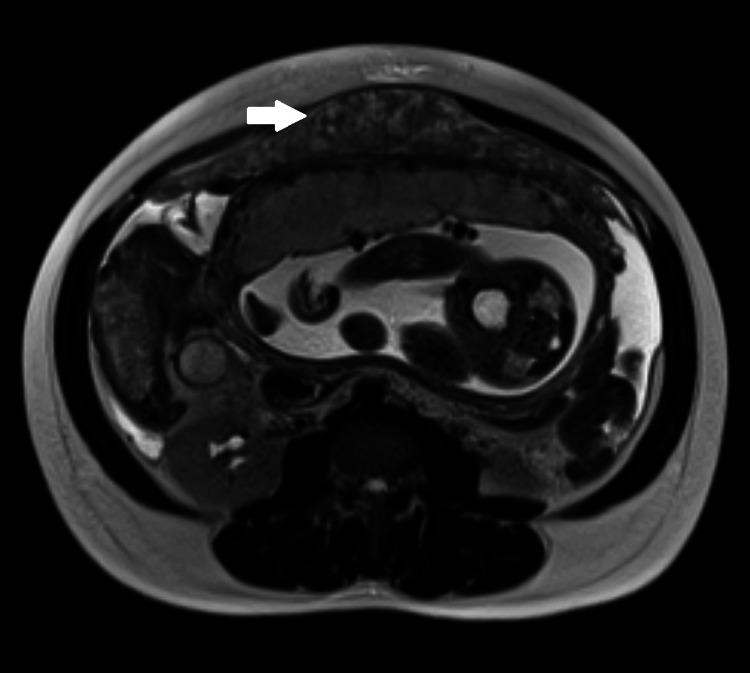
T2-weighted magnetic resonance imaging sequence of the abdomen of a pregnant woman from Haiti. An omental mass of intermediate intensity (white arrow) is shown anterior to the uterus.

Fine-needle aspiration of the omental mass was nondiagnostic. The patient’s vaginal bleeding and abdominal pain persisted, and her cervix dilated. She had an oral temperature of 38.9° Celsius. Exploratory laparotomy demonstrated a friable omental mass with implants on the small bowel; a partial omentectomy was performed at 26 weeks’ gestation. During this procedure, the patient gave birth to a male infant.

Multiple granulomata, some containing acid-fast bacilli, were identified upon histologic examination of the momentum (Technical Appendix Figure). Transcription-mediated amplification of the specimen was positive for *Mycobacterium tuberculosis* rRNA; cultures later grew *M. tuberculosis* susceptible to all first-line antituberculosis medications. Sputum smears and cultures were not performed. The patient’s treatment began with isoniazid, rifampin, ethambutol, and pyrazinamide; her fevers and abdominal pain resolved. Her son was admitted to the neonatal intensive care unit and was placed on antimycobacterial therapy. He also recovered and was discharged after 135 days.

This case highlights several issues related to tuberculosis epidemiology and diagnosis. Although pulmonary disease is the most common manifestation of tuberculosis overall, extrapulmonary tuberculosis accounts for a significant and increasing proportion of cases in the United States (*1*). Pregnancy is associated with greater likelihood of extrapulmonary disease; extrapulmonary infection accounts for 13% of all cases worldwide (*2*) but 50% of cases in pregnancy, according to a recent study (*3*).

The frequency of peritoneal tuberculosis in pregnancy is unknown; few cases have been reported in the literature (*4*–*7*), although we know of 3 additional cases from Haiti (online Technical Appendix Table). However, cases are likely underdiagnosed or diagnosed late in the course of illness. Underdiagnosis and delayed diagnosis may be caused by the nonspecific nature of symptoms, commonly abdominal pain and ascites, which can be attributed to pregnancy itself or obstetrical complications. These erroneous explanations for symptoms are reflected in this patient, whose symptoms were initially attributed to abruption and who was not diagnosed with tuberculosis until >3 weeks after seeking medical assistance. Such delays in diagnosis are typical of peritoneal tuberculosis and are associated with increased death rates (*8*). In many cases, clinical features cannot distinguish peritoneal tuberculosis from malignancy, necessitating more extensive evaluation (*7*).

Failure to diagnose peritoneal tuberculosis, in pregnancy or otherwise, might also stem from the insensitivity of noninvasive diagnostic testing. Paracentesis with acid-fast staining detects only a minority of cases (*8*). The sensitivity of mycobacterial cultures of ascites fluid varies, and culture results are often not available for weeks (*8*). Ascites fluid adenosine deaminase has shown promise as a reliable, minimally invasive diagnostic test in resource-poor countries, but was insensitive in a United States study (*9*). In addition, although tuberculin skin testing and interferon gamma release assay performance are not affected by pregnancy (*10*), neither can distinguish active from latent infection. Without diagnostic clinical features or sensitive noninvasive tests, the diagnosis of peritoneal tuberculosis might only be confirmed through laparoscopy or laparotomy, as in our case. Such invasive testing methods and facilities, equipment, and personnel might not be readily available in resource-poor settings.

This case also illustrates the ongoing threat of tuberculosis in countries of all income levels. It is not clear where our patient contracted tuberculosis; she was most likely exposed in Haiti, but transmission within her Haitian community in the United States, or from another source, is also possible. Regardless, as in her case, a majority of tuberculosis cases within the United States occur in foreign-born persons. Given the ease and frequency of travel, lapses in tuberculosis control in any locale are likely to have effects more broadly. Wherever they work, clinicians must maintain vigilance for tuberculosis in all of its protean forms.

Technical Appendix Case characteristics of peritoneal tuberculosis in pregnancy and diagnostic image.
