# Supplementary material for: Peritoneal Tuberculosis in a Pregnant Woman from Haiti, United States
Source: Emerg Infect Dis. 2013 Mar;19(3):514–6. doi: 10.3201/eid1903.121109 (PMC3647663; doi:10.3201/eid1903.121109)
Supplement: Technical Appendix — Case characteristics of peritoneal tuberculosis in pregnancy and diagnostic image. [file 12-1109-Techapp-s1.pdf]

Article DOI: http://dx.doi.org//10.3201/eid1903.121109

Peritoneal Tuberculosis in a Pregnant Woman from Haiti, United States

Technical Appendix

Technical Appendix Table. Cases of peritoneal tuberculosis in pregnancy from published reports and the authors' clinical experience*

Reference or source	Patient age at presentation, y	Country or region of origin	Symptoms	Duration of illness prior to diagnosis	Diagnostic tests and findings	Pregnancy outcome
Present case	29	Haiti	Vaginal bleeding, abdominal pain, fever	3 wk	MRI of omental mass, granulomata and AFB on omental pathology, positive nucleic acid amplification and omental cultures	Birth at 26 weeks gestation; male infant survived
Case-patient from Haiti #1	20	Haiti	Foul vaginal discharge, abdominal pain, fever	Unknown	Granulomata on placental, uterine, and omental pathology; negative tissue AFB stain	Stillborn child; gestational age unknown; patient had hysterectomy
Case-patient from Haiti #2	26	Haiti	Cough, weight loss, abdominal swelling, fever	Unknown	Clinical diagnosis after response to a therapeutic trial of antitubercular drugs	Birth at 29 weeks gestation; male infant survived
Case-patient from Haiti #3	28	Haiti	Vaginal discharge, abdominal pain, fever	Unknown	Granulomata on placental, uterine, and omental pathology; negative tissue AFB stain	Birth at 28 weeks gestation; female infant died
Coden, 1972 (*1*)	26	Unknown	Abdominal pain, vomiting, anorexia, fever	6 mo	Positive ascites fluid culture for *M. tuberculosis*; therapeutic trial of antitubercular drugs	Term birth; male infant survived
Brooks and Stirrat, 1986 (*2*)	25	Caribbean	Weight loss, nausea, vomiting, fever	Wk, unknown, <1 mo	Ascites fluid AFB stain and culture negative, omental pathology with AFB	Birth at 33 weeks gestation; male infant survived
Lee, 2005 (*3*)	23	South Korea	Ascites, fever	Unknown	Ascites fluid adenosine deaminase 70 U/L, serum CA-125 372 IU/mL, negative ascites fluid AFB stain and culture, MRI with omental cake, granulomata and AFB on omental pathology	Birth at 37 weeks gestation; male infant survived

Reference or source	Patient age at presentation, y	Country or region of origin	Symptoms	Duration of illness prior to diagnosis	Diagnostic tests and findings	Pregnancy outcome
Sakorafas, 2009 (*4*)	28	Ethiopia	Abdominal pain, nausea, vomiting, fever	Mo, unknown	Serum CA-125 163 IU/mL, ascites fluid AFB stain and culture negative, granulomata on peritoneal pathology, peritoneal biopsy cultures positive for *M. tuberculosis*	Pregnancy without other complications; otherwise, details unknown

* Published reports are limited to those in English. MRI, magnetic resonance imaging; AFB, acid-fast bacilli.

References

1. Coden J. Tuberculous peritonitis in pregnancy. BMJ. 1972;3:153. PubMed http://dx.doi.org/10.1136/bmj.3.5819.153

2. Brooks JH, Stirrat GM. Tuberculous peritonitis in pregnancy. Case report. Br J Obstet Gynaecol. 1986;93:1009–10. PubMed http://dx.doi.org/10.1111/j.1471-0528.1986.tb08027.x

3. Lee GS, Kim SJ, Park IY, Shin JC, Kim SP. Tuberculous peritonitis in pregnancy. J Obstet Gynaecol Res. 2005;31:436–8. PubMed http://dx.doi.org/10.1111/j.1447-0756.2005.00316.x

4. Sakorafas GH, Ntavatzikos A, Konstantiadou I, Karamitopoulou E, Kavatha D, Peros G. Peritoneal tuberculosis in pregnancy mimicking advanced ovarian cancer: a plea to avoid hasty, radical and irreversible surgical decisions. Int J Infect Dis. 2009;13:e270–2. PubMed http://dx.doi.org/10.1016/j.ijid.2008.11.003

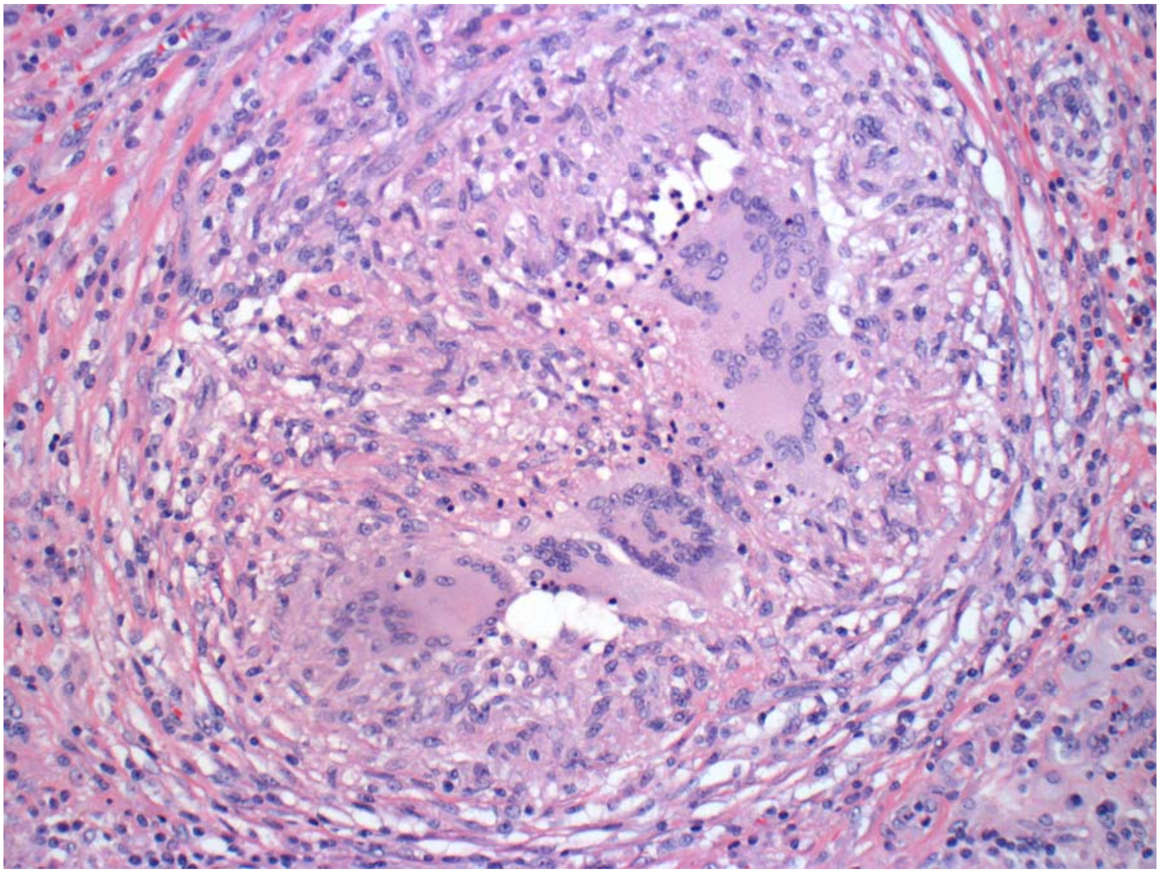

Technical Appendix Figure. Hematoxylin and eosin–stained tissue from the patient’s omental biopsy, demonstrating necrotizing granulomata. Acid-fast stains of the specimen showed rare acid-fast bacilli. Original magnification ×400.
